# The structure of the S-layer of *Clostridium difficile*

**DOI:** 10.1007/s12079-017-0429-z

**Published:** 2017-11-23

**Authors:** William J. Bradshaw, April K. Roberts, Clifford C. Shone, K. Ravi Acharya

**Affiliations:** 1 Department of Biology and Biochemistry, University of Bath, Claverton Down, Bath, BA2 7AY UK; 2 Public Health England, Porton Down, Salisbury, SP4 0JG UK

**Keywords:** *Clostridium Difficile*, S-layer, *C. difficile* Infection, Cell wall protein, Bacterial adhesion, Colitis

## Abstract

The nosocomially acquired pathogen *Clostridium difficile* is the primary causative agent of antibiotic associated diarrhoea and causes tens of thousands of deaths globally each year. *C. difficile* presents a paracrystalline protein array on the surface of the cell known as an S-layer. S-layers have been demonstrated to possess a wide range of important functions, which, combined with their inherent accessibility, makes them a promising drug target. The unusually complex S-layer of *C. difficile* is primarily comprised of the high- and low- molecular weight S-layer proteins, HMW SLP and LMW SLP, formed from the cleavage of the S-layer precursor protein, SlpA, but may also contain up to 28 SlpA paralogues. A model of how the S-layer functions as a whole is required if it is to be exploited in fighting the bacterium. Here, we provide a summary of what is known about the S-layer of *C. difficile* and each of the paralogues and, considering some of the domains present, suggest potential roles for them.

## Introduction

*Clostridium difficile* is a rod-shaped, obligate anaerobic, Gram-positive, spore-forming bacterium. The bacterium is usually nosocomially acquired and only pathogenic after disruption of the gut flora, primarily through the use of antibiotics. *C. difficile* infection (CDI) can result in mild to severe diarrhoea, colitis, pseudomembranous colitis, toxic megacolon and, ultimately, death (Kachrimanidou and Malisiovas 2011). Thirty-day mortality rates have been shown to be over 30% (McGowan et al. 2011). CDI causes tens of thousands of deaths globally each year and treatment costs billions of dollars (Kachrimanidou and Malisiovas 2011; Scott 2009; Wiegand et al. 2012). There has also been a significant global increase in *C. difficile* antibiotic resistance since the early 1990s, which has led to more cases, greater morbidity and mortality and ever increasing costs (Barkin et al. 2017; Kachrimanidou and Malisiovas 2011; Ong et al. 2017). This presents a clear need for greater understanding of *C. difficile* to facilitate the development of new methods of fighting the disease. To this end, the surface layer (S-layer) of *C. difficile*, which was first identified by Kawata et al. (1984), has received considerable attention over the last 15 years.

S-layers have been observed in hundreds of prokaryotic species, including a diverse range of bacteria and virtually all archaea. A typical S-layer consists of a single protein arranged in a two dimensional paracrystalline array, forming the outermost surface of the cell (Sara and Sleytr 2000; Smarda et al. 2002). An S-layer may allow the surface presentation of other proteins anchored deeper in the cell wall, but will, by far, form the majority of the externally presented cell surface (Desvaux et al. 2006).

S-layer proteins can account for 15% of the total protein of a cell (Sara and Sleytr 2000), and their need for continuous replenishment necessitates the translation of around 500 molecules per second (Smarda et al. 2002). It can be inferred from the high metabolic cost of having an S-layer that it must fulfil significant and essential requirements of the cell. Many important S-layer functions have been demonstrated, they include, but are not limited to: archaeal cell shape determination, molecular sieving, the degradation, transport or storage of nutrients or proteins involved in the same, host cell adhesion and/or invasion, immune system evasion, and protection from competing microorganisms (Sara and Sleytr 2000).

## *Clostridium difficile* S-layer

**Fig. 1:**
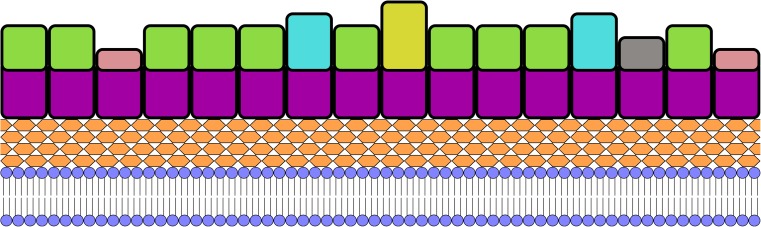
Schematic diagram of the S-layer of *C. difficile*. The lipid bilayer is shown in blue with the peptidoglycan in peach. Above this is a purple layer formed by the three cell wall binding domains of HMW SLP and paralogues. The surface exposed “functional” regions are shown on top, the majority of which are LMW SLP, shown in green. The S-layer also contains other proteins with a range of functions

Unlike the majority of S-layers, which consist of a single protein, the mature S-layer of *C. difficile* is largely heterodimeric but may contain over 30 other proteins (Fagan et al., 2011b; Monot et al. 2011; Sebaihia et al. 2006). The majority of the S-layer is formed by the low and high molecular weight S-layer proteins (LMW SLP and HMW SLP - previously known as P36 and P47, respectively), which are coded for by a single gene: *slpA* (Calabi et al. 2001; Karjalainen et al. 2001). HMW SLP is formed of three putative cell wall binding domains (CWBDs – Pfam 04122, CWB2) (Fagan and Fairweather 2014; Fagan et al. 2011b; Monot et al. 2011; Sebaihia et al. 2006), while the fold of LMW SLP is potentially unique to *C. difficile* (Fagan et al. 2009). The two proteins form a heterodimer on the surface of the cell (Fagan et al. 2009) with HMW SLP forming a lower layer and LMW SLP forming an upper, surface exposed layer (Fig. 1) (Cerquetti et al. 2000). Despite indications to the contrary from early studies (Cerquetti et al. 2000; Cerquetti et al. 1992; Mauri et al. 1999), the S-layer of *C. difficile* does not appear to normally be glycosylated (Qazi et al. 2009), although a glycosylation gene cluster has been identified in some strains (Dingle et al. 2013).

Using modified bacteriocins – bacterial proteins that resemble a bacteriophage tail capable of forming pores and depolarising competing bacterial cells – Kirk et al. (2017) recently identified two *C. difficile* strains that lack an S-layer and were therefore, not susceptible to the bacteriocins used. These strains showed significantly increased susceptibility to lysozyme and the antimicrobial peptide LL-37, an inability to produce symptoms of CDI in hamsters and decreased toxin release. They also showed a reduction in spore production, viability and heat resistance. This demonstrates the importance of the S-layer in a range of processes but also that it appears not to be absolutely essential to the survival of the bacterium.

**Fig. 2:**
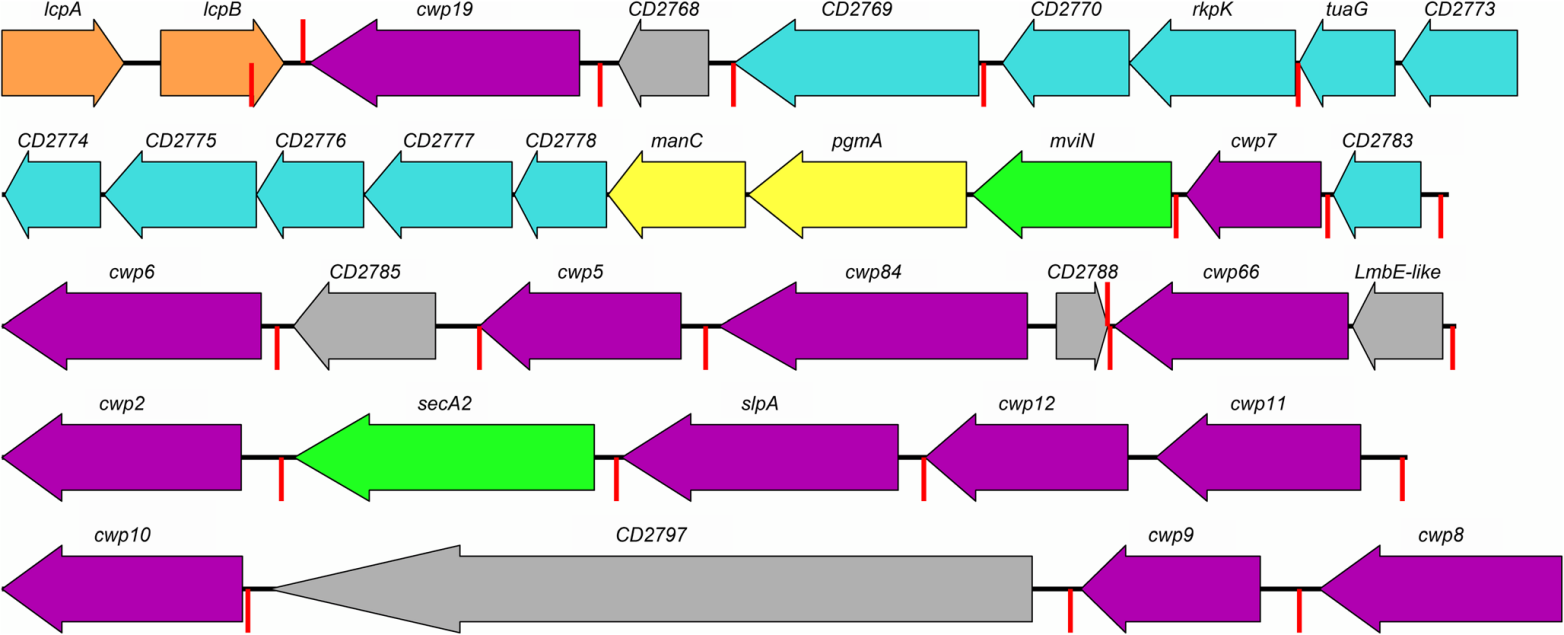
The AP and *slpA* loci. The two adjacent loci, which respectively code for proteins involved in the production of PSII and proteins that attach to PSII, are shown. Genes coding for proteins with CWB2 domains are shown in purple, those involved in polysaccharide metabolism in cyan, attachment to peptidoglycan in peach, mannose biosynthesis in yellow and biopolymer export in green, other functions are in grey. *CD2768* – hydrolase, *CD2785* – membrane protein, *CD2788* – GtrA-like membrane protein, *CD2797* – calcium binding adhesin. Terminators predicted by Genome2D (Baerends et al. 2004) are shown in red

**Fig. 3:**
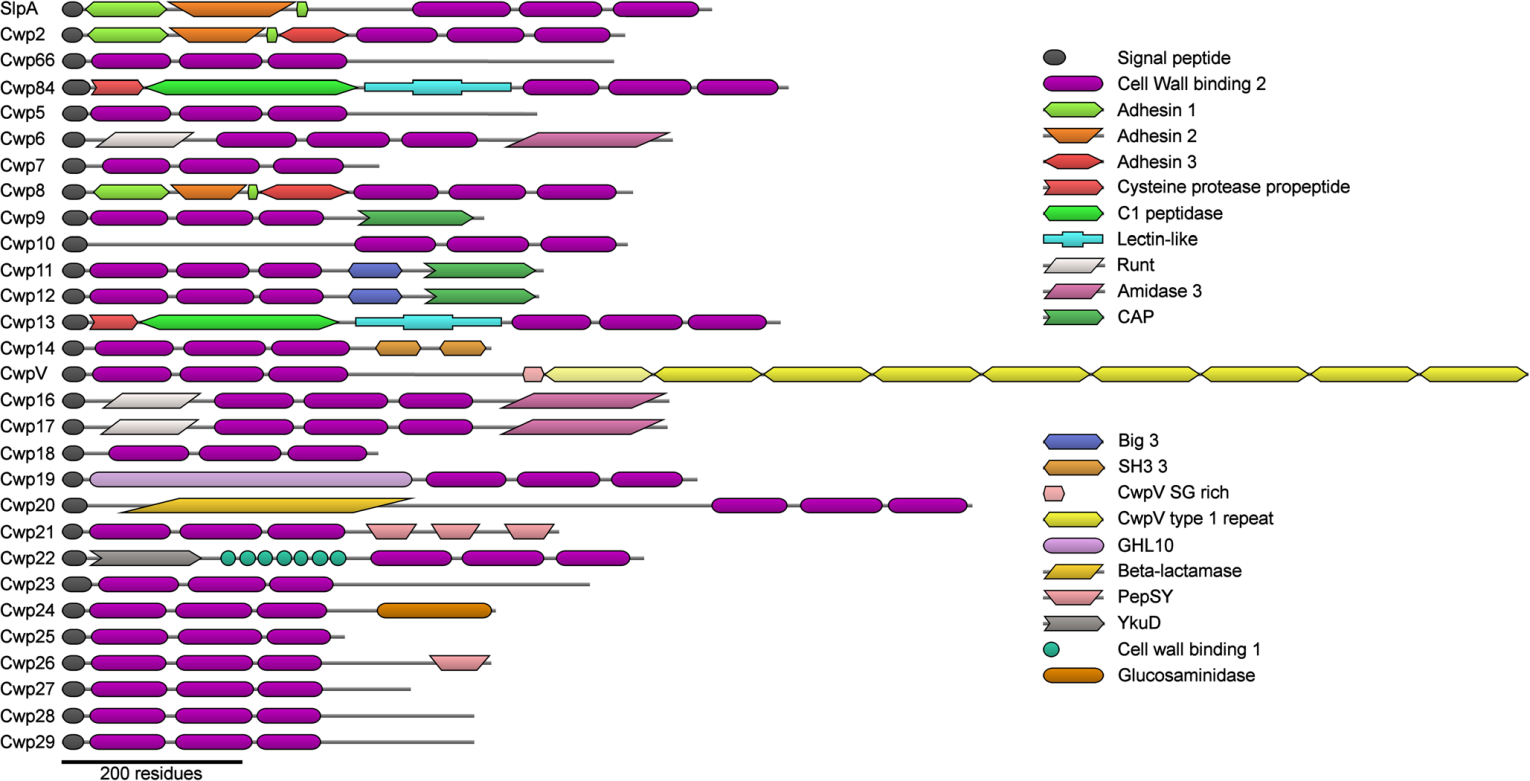
Putative domain representation of the 29 *cwp* genes found in the *Clostridium difficile* 630 genome. Each codes for three cell wall binding domains, while all except *cwp18*, *cwp25*, and potentially *cwp7* appear to code for at least one other domain, which is likely to confer a specific function on the protein. Generated using DoMosaics with HMMER (Eddy 2008; Moore et al. 2014)

*slpA* sits in a 36.6 kb (strain 630) region of the *C. difficile* genome, known as the *slpA* locus. This locus contains 11 *slpA* paralogs (Fig. 2) and there are 17 more paralogs scattered throughout the genome (Fagan et al. 2011b; Monot et al. 2011; Sebaihia et al. 2006). All of these genes code for a protein with an N-terminal signal peptide and three putative cell wall binding domains with significant similarity to HMW SLP (Calabi et al. 2001; Karjalainen et al. 2001). These paralogs are known as “cell-” or “clostridial wall proteins”, or more commonly by the abbreviated form “CwpX” (X = 1–29). Four *cwp*s (*slpA*, *cwp66*, *cwp84* and *cwpV*) were characterised and named before this convention was established (Fagan et al. 2011b). As well as the characteristic three cell wall binding domains, most Cwps also possess at least one other domain, allowing the *C. difficile* S-layer the potential to possess an unusually wide range of functions (Fig. 3). Many of the Cwps are, however, yet to be characterised in any significant way, meaning that an encompassing model of the structure and functions of the S-layer is yet to be established. The intrinsic importance of S-layers combined with their inherent accessibility and the apparent complexity of the S-layer of *C. difficile* may therefore yield a plethora of information that could be exploited in future drug development.

Many of the genes within the *slpA* locus show significant variation between strains, particularly in areas that code for the surface exposed “functional” regions. *slpA*, *cwp66*, and *secA2*, which are almost contiguous and appear to be able to undergo horizontal transfer as a group alongside *cwp2*, have been noted as having particularly high variation for genes within the *slpA* locus (Dingle et al. 2013). The functional region of *cwp66* has been observed as having as little as 33% identity between strains (Karjalainen et al. 2001). A variant of the slpA locus has been identified that lacks *cwp2*, which is replaced by a 23.8 kb predicted S-layer glycosylation gene cluster (Dingle et al. 2013). It has also been demonstrated that, at the very least, strain 630 expresses the first seven Cwps (Calabi et al. 2001) and presents Cwp2, Cwp84, Cwp6, Cwp12, CwpV, Cwp24 and Cwp25 on the surface of the cell under normal growth conditions (Wright et al. 2005). Interestingly, despite their expression, Cwp66 and Cwp5 were not present in cell surface extracts.

As well as containing the first 12 of the 29 *cwp* genes, the *slpA* locus also contains 6 other genes: 2 putative membrane proteins of unknown function, a putative LmbE-like deacetylase, a non-redundant accessory Sec gene, a putative calcium-binding adhesion protein, and a putative glycosyltransferase (Fig. 2) (Monot et al. 2011; Sebaihia et al. 2006). The accessory Sec gene - *secA2* - has been demonstrated to be necessary for the secretion of at least some Cwps (Fagan and Fairweather 2011), although there is a significant possibility that it is required for all of them, and it has been suggested that each of the non-*cwp* genes within the *slpA* locus may be involved in cell wall synthesis (Calabi et al. 2001).

Biazzo et al. (2013) analysed 14 of the other 17 *cwp* genes scattered throughout the *C. difficile* genome; amplification of *cwp14*, *cwp21*, and *cwp23* was unsuccessful, so they were excluded from the study. They observed that *cwp13*, *cwpV* (with the exception of the repeat regions, discussed later), *cwp16*, *cwp18*, *cwp19*, *cwp20*, *cwp22*, *cwp24* and *cwp25* have well conserved sequences and expression, suggesting that they may possess important functions. *cwp17*, *cwp26*, *cwp27*, *cwp28*, and *cwp29* tended to be less conserved with considerable variation in expression levels between ribotypes, even when the genes possessed identical sequences (Biazzo et al. 2013). This, along with the fact that *cwp27*, *cwp28*, and *cwp29* are not present in certain ribotypes, suggests that these genes may possess less important functions.

To develop a full model of the workings of the S-layer, a thorough understanding of the role of each protein is required. Here we provide a discussion of what is known about each protein and the potential role(s) of their functional domains. Each protein can be compared to its schematic in Fig. 3 as a reference.

## SlpA

SlpA is the primary component of the *C. difficile* S-layer and is usually by far the most abundant constituent of cell surface extracts (Ferreira et al. 2017; Wright et al. 2005). It is cleaved after secretion to produce two proteins: HMW SLP and LMW SLP, which form the heterodimeric “H/L complex” (Fagan et al. 2009), this polymerises to form the mature S-layer. HMW SLP binds to the cell wall through a non-covalent interaction (Willing et al. 2015), while LMW SLP is presented as the outermost surface of the cell (Calabi et al. 2001). LMW SLP can be extracted from *C. difficile* through relatively gentle methods while the removal of HMW SLP requires more harsh conditions (Wright et al. 2005).

The cell wall binding domains of HMW SLP and other Cwps bear low homology to LytB and LytC, two proteins from *Bacillus subtillis* (Calabi et al. 2001). LytB is an N-acetylmuramic acid L-alanine amidase, also known as a peptidoglycan amidohydrolase, while LytC modulates its activity and may too possess amidase activity (Lazarevic et al. 1992). HMW SLP exhibits some amidase activity (Calabi et al. 2001), but it is unknown if this function is related to cell wall synthesis or binding or if the CWB2 domains in other Cwps also possess amidase activity. N-acetylmuramic acid L-alanine amidases have also been shown to bind teichoic acids, polysaccharides embedded in bacterial cell walls (Herbold and Glaser 1975; Lazarevic et al. 1992).

Despite a high level of variability in the SlpA gene (Dingle et al. 2013), including HMW SLP having a mass between 41 and 48 kDa (Calabi et al. 2001), antibodies raised against HMW SLP from one ribotype retain activity against HMW SLP from another (Cerquetti et al. 2000; Karjalainen et al. 2001). LMW SLP, on the other hand, which is considerably more variable than HMW SLP, can have a mass ranging from 32 to 38 kDa and has no significant similarity to any other proteins (Calabi et al. 2001). LMW SLP is not always recognised by antibodies raised against another ribotype. This variability is likely to have arisen in an attempt to evade the host immune system (Calabi et al. 2001; Cerquetti et al. 2000; Spigaglia et al. 2011), which is also likely to be the reason why other Cwps show an increased level of variability between strains (Dingle et al. 2013). This variability has also been shown to be likely to result in variations in adhesion of *C. difficile* to mammalian cells, suggesting a role for LMW SLP in host cell adhesion (Merrigan et al. 2013).

**Fig. 4:**
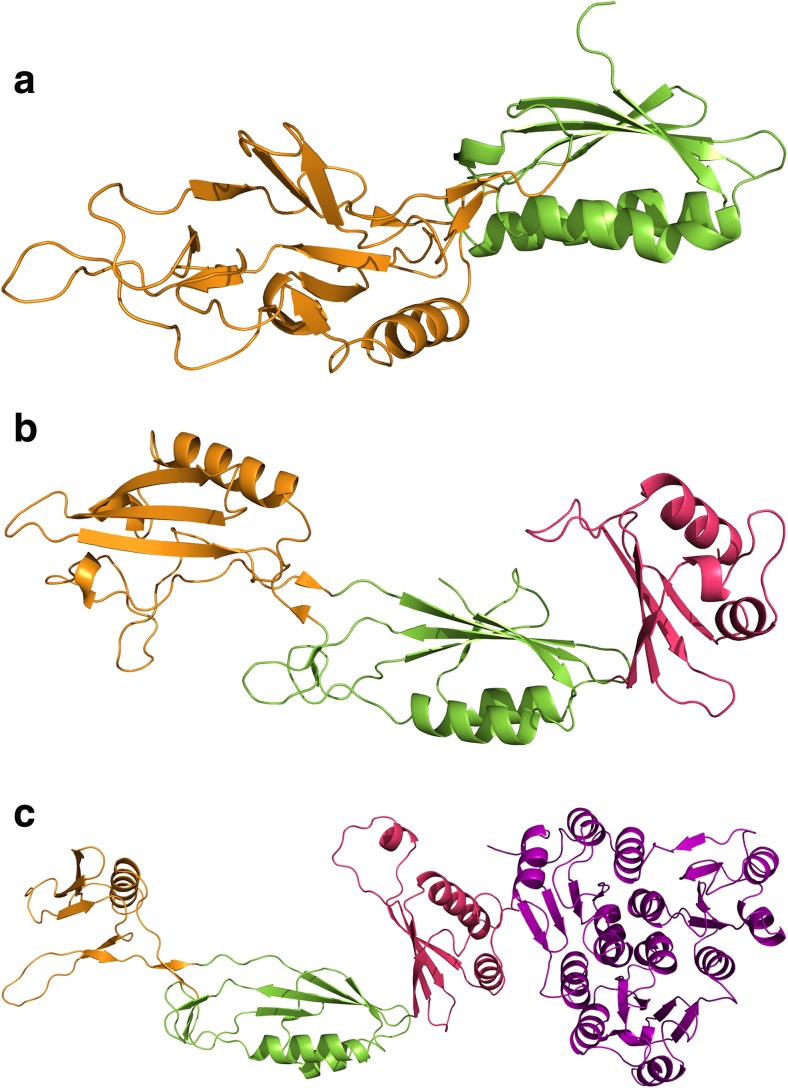
Adhesin structures. **a** LMW SLP: 3CVZ (Fagan et al. 2009). **b** Cwp2: 5NJL (Bradshaw et al. 2017a). **c** Cwp8: 5J7Q (Usenik et al. 2017). Cwp2 and Cwp8 assume similar folds with domain 2 rotated approximately 40°. Domain 2 of LMW SLP has significantly longer loop regions and is positioned differently to that of Cwp2 and Cwp8. LMW SLP is covalently bound to HMW SLP so it is likely that domain 3 of LMW SLP is at least somewhat different to that of Cwp2 and Cwp8. Domain colours follow those given in Fig. 3

The first insights into the structure of the S-layer of *C. difficile* were obtained by Cerquetti et al. (2000) who used two different methods to visualise the S-layer by scanning electron microscopy. This demonstrated that two separate layers are formed, a lower one with apparent hexagonal symmetry formed by HMW SLP and an upper one with apparent square symmetry formed by LMW SLP. These images, however, did not yield additional structural detail beyond determination of the symmetry of the S-layer. Fagan et al. (2009) analysed the structure of the H/L complex using small angle X-ray scattering (SAXS) and determined the crystal structure of a fragment of LMW SLP at 2.4 Å (PDB: 3CVZ, Fig. 4a). This structure was of a truncated form missing 59 residues from the C-terminus that are likely to be involved in formation of the H/L complex. The N and C-terminal regions form a small two-layer sandwich, while the central region assumes a novel fold consisting of around 60% loops. As this domain is likely to be surface exposed, it was suggested that the loops allowed for the high level of sequence variability that confers immune system evasion (Spigaglia et al. 2011), while retaining the overall fold (Fagan et al. 2009). The majority of this variation is seen in domain 2, which is likely to be the most surface exposed domain and therefore play a primary role in adhesion, explaining the previously observed variations in adhesive properties between strains (Merrigan et al. 2013).

Using Cwp2 and Cwp66, it was demonstrated that the three cell wall binding domains present in HMW SLP and all other Cwps mediate attachment to the cell surface through an interaction with PSII, a surface bound teichoic acid-like polymer formed of a repeating hexasaccharide-phosphate (Ganeshapillai et al. 2008; Willing et al. 2015). It was also demonstrated that, despite their similarity, the three CWB2 domains are not redundant - each is required for binding to PSII. Removing individual domains, replacing them with a second copy of another or altering their order prevents binding to the cell wall. The AP locus, immediately upstream of the slpA locus (Fig. 2) has been shown to be responsible for the synthesis and export of PSII (Chu et al. 2016; Willing et al. 2015).

Willing et al. (2015) also claimed that binding to PSII is mediated through a conserved Pro, Ile/Leu/Val, Ile/Leu/Val, Ile/Leu/Val or “PILL” motif. Although the same method of binding is very likely to be used by all Cwps, it has been demonstrated that different methods of S-layer extraction will yield different combinations of Cwps, suggesting slight variations on binding mechanism or strength (Wright et al. 2005).

The PILL motif was, however, recently shown not to be responsible for binding to PSII with the publication of full length structures of Cwp6 (PDB: 5 J72) and Cwp8 (PDB: 5J6Q) (Usenik et al. 2017). The CWB2 domains each assume a toprim (topoisomerase-primase) fold, together forming a trefoil-like shape. Trimerisation is achieved through the PILL motif with PSII binding mediated by residues across all three domains, explaining why the domains are not interchangeable (Usenik et al. 2017; Willing et al. 2015).

## Cwp2 and Cwp8

We have recently demonstrated that a *cwp2* knockout has impaired adhesion to mammalian cells in vitro. This demonstrates a potential role for Cwp2 in host cell adhesion (Bradshaw et al. 2017a). This was accompanied by the structure of the functional region of Cwp2 (PDB: 5NJL, Fig. 4b), which assumes an extended three domain fold. Despite no significant sequence identity, domains 1 and 2 bear significant similarity to the equivalent domains from LMW SLP, although the loop regions in domain 2 are much shorter in Cwp2. It is therefore likely that the two proteins use similar methods of adhesion. The full length structure of Cwp8 (PDB: 5J6Q, Fig. 4c) was also recently determined (Usenik et al. 2017). The functional region bears a high degree of similarity to that of Cwp2, however domain 2 appears to be rotated approximately 40°, although a hinge region is present in all three proteins allowing a degree of movement (Bradshaw et al. 2017a; Usenik et al. 2017). Based on the similarity between the proteins, it is highly likely that Cwp8 also has adhesive properties. The effect that variation in domain 2 between LMW SLP, Cwp2 and Cwp8 has on adhesion is yet to be characterised.

## Cwp66

Due to a low but significant level of similarity to known bacterial adhesins, Waligora et al. (2001) predicted that Cwp66 could also be an adhesin and analysed its ability to perform this function. They observed that Cwp66 is secreted under normal growth conditions and that surface presentation is increased in response to heat-shock. Adherence to Vero cells by heat-shocked *C. difficile* was partially abrogated by antibodies raised against Cwp66 – particularly those raised against the likely surface exposed C-terminal functional region, but it was not affected without prior heat-shocking (Waligora et al. 2001). The functional region of Cwp66, which bears no significant similarity to any previously determined folds (Altschul et al. 1990), contains three imperfect 21–23 residue repeats and is predicted to assume a structure mostly comprised of β-strands (Kelley et al. 2015; Slabinski et al. 2007; Waligora et al. 2001).

*cwp66* is located 32 bp (strain 630) downstream of the putative LmbE-like deacetylase gene also found in the *slpA* locus (Fig. 2). The two genes have no separating terminator or promoter, so are polycistronically co-transcribed. The LmbE-like superfamily consists of a wide range of metallohydrolases, the majority of which bind zinc as a cofactor. All members of the family possess a Rossmann fold and cleave substrates containing an N-acetylglucosamine moiety. Many LmbE-like proteins have been shown to possess cell wall related functions, so the family is of particular interest for drug development (Viars et al. 2014).

## Cwp84 and Cwp13

Cwp84 and Cwp13 each possess a C1A cysteine protease domain (also known as a papain protease domain). Cwp84 is responsible for the cleavage of SlpA to form HMW SLP and LMW SLP (Dang et al. 2010; Kirby et al. 2009). It has also been shown to be capable of breaking down gelatine and the extra cellular matrix proteins fibronectin, laminin, and vitronectin, but is unable to cleave type IV collagen (Janoir et al. 2004; Janoir et al. 2007). Cwp84 knockouts present full length SlpA on the surface of the cell. This results in an abnormal S-layer and the presence of SlpA, Cwp2 and Cwp66 in growth medium, which is not seen in the wild type (Kirby et al. 2009). Knockouts also show aberrant colony morphology, grow at half their usual rate, and have a propensity to aggregate (de la Riva et al. 2011; Kirby et al. 2009). A Cwp84 knockout strain was, however, still able to cause CDI in hamsters (Kirby et al. 2009), but it has been suggested that perturbation of S-layer formation may make the bacterium more susceptible to antibiotics (Dang et al. 2010).

Despite a high level of identity to Cwp84, Cwp13 appears to possess different functions and is not as essential to correct functioning of the cell (de la Riva et al. 2011). While Cwp84 cleaves SlpA between LMW SLP and HMW SLP, Cwp13 cleaves it within one of the cell wall binding domains, rendering the protein useless. It has been speculated that this function may facilitate the removal of misfolded protein, ensuring a fully functional S-layer (de la Riva et al. 2011).

Papain proteases possess an N-terminal propeptide and are frequently, but not always, able to autoactivate (Beton et al. 2012; ChapetonMontes et al. 2011; Dahl et al. 2001; Nagler et al. 1999). Cwp84 is unlikely to be capable of autoactivation, while Cwp13 is likely to possess this ability. Cwp13 has also been shown to be capable of removing the propeptide from Cwp84, although it does not appear to be entirely responsible for this as Cwp13 knockouts present both the proenzyme and mature Cwp84 (de la Riva et al. 2011).

**Fig. 5:**
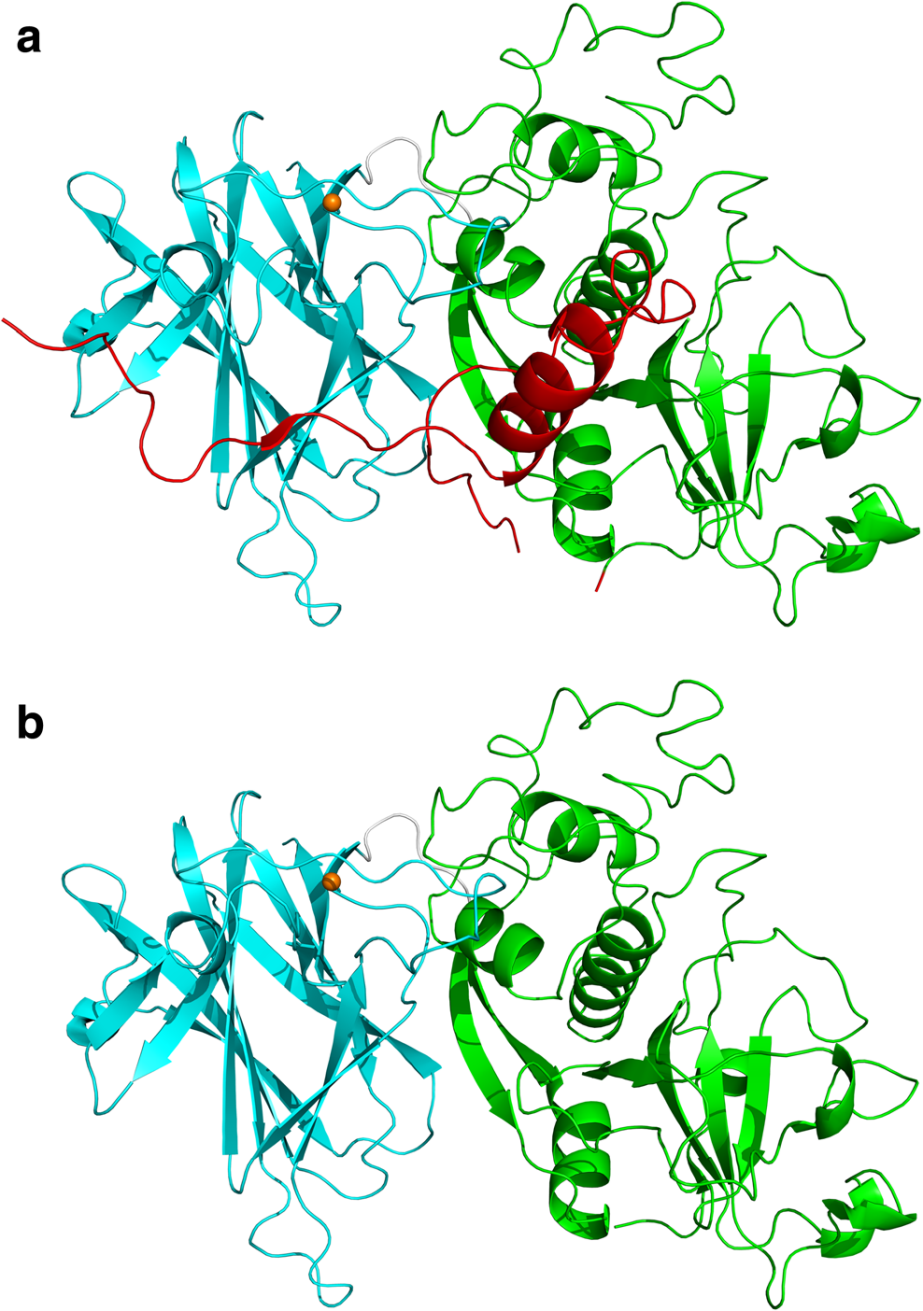
The structure of Cwp84. **a** With the propeptide: 4CI7 (Bradshaw et al. 2014), **b** Without the propeptide: 4D59, 4D5A (Bradshaw et al. 2015). Cwp84 possesses a cysteine protease domain with a cathepsin L-like fold and a “lectin-like” domain of currently unknown function that closely interacts with the cysteine protease domain forming part of the active site groove. Domain colours follow those given in Fig. 3

We have determined the structure of the functional region of Cwp84, both with the propeptide (PDB: 4CI7, Fig. 5a) and without (PDB: 4D5A, 4D59, Fig. 5b). The cysteine protease domain exhibits a cathepsin L-like fold and is separated from the cell wall binding domains by a “lectin-like” domain of currently unknown function (Bradshaw et al. 2014). Upon cleavage of the propeptide, Cwp84 undergoes slight conformational changes, which potentially allow SlpA to bind and expose a hydrophobic pocket on the surface of the lectin like domain (Bradshaw et al. 2015).

## Cwp6, Cwp16 and Cwp17

Unlike the rest of the family, which possess either N- or C-terminal cell wall binding domains, those of Cwp6, Cwp16 and Cwp17 are central within the protein rather than at either of the termini. The three proteins have been predicted to possess an amidase 3 domain at the C-terminus, while no structure was able to be predicted for a region of approximately 150 residues at the N-terminus (Eddy 2008). The effect that the positioning of the cell wall binding domains, whether N-terminal, C-terminal, or indeed, central, has on the overall structure of Cwps, and their positioning relative to the cell wall and therefore their interactions with PSII is unknown.

**Fig. 6:**
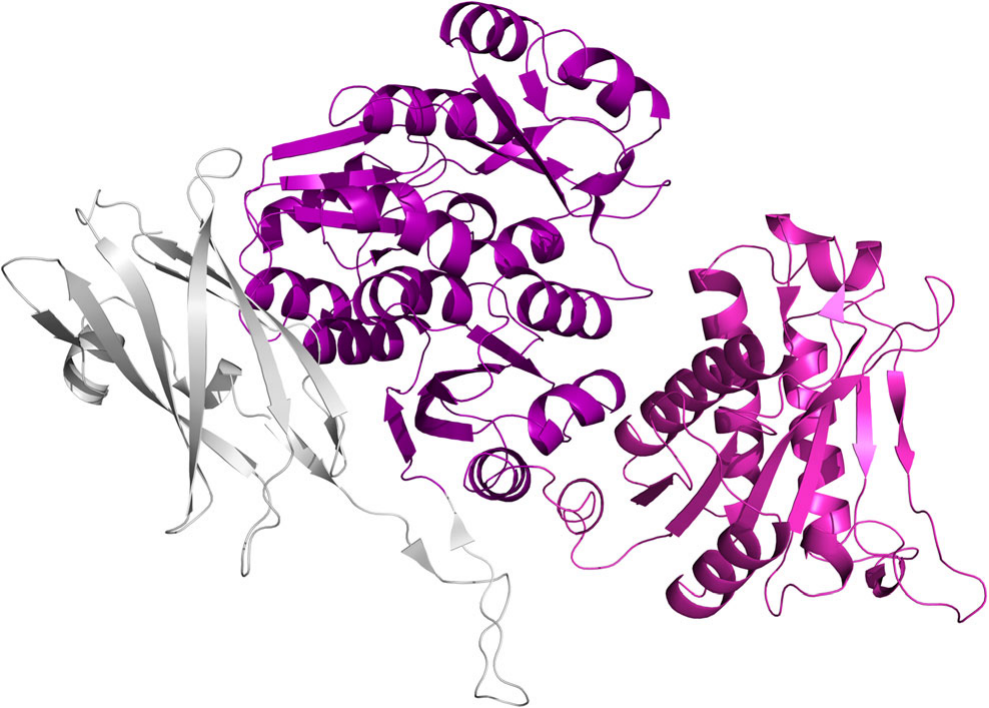
The structure of Cwp6. 5J72 (Usenik et al. 2017). Cwp6 possesses central cell wall binding domains flanked by a Runt domain and an amidase 3 domain. The function of the usually metazoan runt domain is currently unknown. Domain colours follow those given in Fig. 3

The recently determined structure of Cwp6 (PDB: 5 J72, Fig. 6) confirmed the predicted C-terminal amidase domain and showed the presence of a seven-stranded β-barrel at the N-terminus, which is also likely to be present in Cwp16 and Cwp17 (Usenik et al. 2017). The β-barrel bears a high level of structural similarity to the runt homology domain from the RUNX family of eukaryotic transcription factors. The RUNX family of proteins are a group of metazoan transcription factors whose functions can be modulated via a wide range of posttranslational modifications and have been shown to be frequently downregulated in cancer (Ito et al. 2015). Heterodimeric RUNX proteins appear to act as weak transcriptional repressors on their own, but when complexed with other proteins can act as considerably stronger activators or repressors (Durst and Hiebert 2004). It does not appear that prokaryotic RUNX domains have been previously observed, so the role of this domain in Cwp6, Cwp16 and Cwp17 is unclear. As the eukaryotic domains are involved in a significant number of protein-protein interactions, this may also be the case in prokaryotes.

Amidase 3 domains possess N-acetylmuramic acid L-alanine amidase activity – that is to say they are capable of cleaving the bond between N-acetylmuramic acid and L-alanine in peptidoglycan crosslinks (Senzani et al. 2017). The knockout of an Amidase 3 containing protein from *Mycobacterium smegmatis* recently showed impaired cell division, increased susceptibility to antibiotics and increased cell permeability (Senzani et al. 2017). An ability to break down peptidoglycan was demonstrated for Cwp6 (Usenik et al. 2017), however, as previously noted, HMW SLP has also been shown to possess amidase activity (Calabi et al. 2001). Whether the amidase activity shown by Cwp6 is conferred by the amidase 3 domain, the cell wall binding domains, or both was not considered.

## Cwp9, Cwp11 and Cwp12

The N-terminal cell wall binding domains of Cwp12 are followed by a type 3 bacterial immunoglobulin-like domain (Big 3) and a CAP domain (Eddy 2008) (named after the related mammalian Cysteine-Rich Secretory Proteins, insect Antigen 5 proteins, and plant Pathogenesis-Related proteins) (Gibbs et al. 2008). Despite bearing 63% identity and 80% similarity to Cwp12 (Altschul et al. 1990), a Big 3 domain is not detected in Cwp11 by an HMM search (Eddy 2008). This is likely to be due to the low sequence similarity frequently seen in Big 3 domains (Bateman et al. 1996). Cwp9 is approximately 75 residues shorter as it does not contain a Big 3 domain.

Bacterial immunoglobulin-like domains (Big domains), which are likely to have evolved either divergently or horizontally from their eukaryotic cousins (Bateman et al. 1996) are frequently found on the surface of bacterial cells (Wang et al. 2013) and have been shown to be involved in host cell adhesion and invasion (Czibener and Ugalde 2012; Hamburger et al. 1999; Luo et al. 2000). Despite particularly low sequence similarity, all members of the family have been predicted to have largely similar structures (Bateman et al. 1996; Wang et al. 2013).

The first structure of a Big 3 domain, that of *Streptococcus pneumoniae* SP0498, was published in 2013 (Wang et al.). Big 3 domains consist of an eight stranded stretched β-barrel, a somewhat different structure to that of eukaryotic immunoglobulins, which possess more of a β-sandwich (Wang et al. 2013). SP0498 was demonstrated to be a calcium binding protein, a feature that is potentially common to all Big domains (Raman et al. 2010). It was speculated that calcium binding is important to the role of Big domains in host cell adhesion and invasion (Wang et al. 2013).

In eukaryotes, CAP domains are involved in a wide range of signalling processes and their roles have been studied extensively. Members of the superfamily have an α-β-α sandwich fold and appear to function through a conserved “incomplete protease” active site containing two histidine residues and an acidic residue (usually glutamate) (Gibbs et al. 2008). The wide range of functions exhibited by proteins possessing CAP domains is often conferred through another domain or a C-terminal extension (Brangulis et al. 2015).

Despite being widespread, prokaryotic CAP domains are yet to be as extensively characterised as their eukaryotic homologues. Brangulis et al. (2015) determined the structure of BB0689, a surface presented CAP domain from *Borrelia burgdorferi* that has a potential role in pathogenesis, and performed a range of assays to identify the function of the protein. The study showed that bacterial CAP domains possess the conserved features seen in eukaryotic CAPs, but was unable to identify any function.

## Cwp14

Cwp14 contains N-terminal cell wall binding domains and two domains that are classified by Pfam as bacterial SH3 domains, also known as type 3 SH3 domains (Finn et al. 2016), while InterPro classifies the domains as SH3-like domains (Jones et al. 2014).

SH3 (Src Homology 3) domains, named after the Rous Sarcoma Virus tyrosine kinase, v-Src (Thomas and Brugge 1997), to which they have significant sequence similarity are 50–60 residue domains that form a 5 or 6 stranded beta sandwich with a hydrophobic ligand binding pocket capable of binding proteins with a PXXP motif. The domain facilitates a wide range of protein-protein interactions across all organisms and is has a very large range of functions (Mayer 2001; Weng et al. 1995).

## CwpV

CwpV has N-terminal cell wall binding domains followed by a region of approximately 200 residues of unknown structure and function, a short Ser/Gly rich region, and several repeat regions. The sequence of CwpV is very well conserved between ribotypes up to and including the Ser/Gly rich region (Reynolds et al. 2011). The protein has been shown to mediate cell aggregation and phage resistance. Overexpression results in smaller, more densely packed colonies and decreased susceptibility to infection by bacteriophages, while knocking down or knocking out results in larger, sparser colonies and increased susceptibility (Reynolds et al. 2011; Sekulovic et al. 2015). The mechanisms by which CwpV causes aggregation and phage resistance are currently unknown, however, two particularly interesting features have been observed: firstly, the level of expression is controlled by phase variability of the gene (Emerson et al. 2009), secondly, the repeat regions are highly variable but appear to retain their function (Reynolds et al. 2011).

CwpV is expressed by 0.1–10% of *C. difficile* cells, regardless of descent from a common parent cell and accounts for approximately 13% of the S-layer (Reynolds et al. 2011). Expression is controlled by the recombinase RecV, which inverts a pair of imperfect inverted repeat regions located between the promoter and the start codon. This results in two possible mRNA transcripts, one that results in translation of CwpV (termed “ON”), and one that does not (“OFF”). The differences between the two transcripts result in the formation of a stable stem loop intrinsic terminator structure in the OFF transcript that is not formed in the ON transcript. When RNA polymerase reaches the intrinsic terminator, transcription is stopped and the complex destabilised, preventing transcription (Emerson et al. 2009). RecV has also been shown to control a “flagellar switch” in the same way as CwpV. This results in the presentation of flagella in the ON orientation and a lack of flagella in the OFF position. One of the genes controlled by the flagellar switch is *sigD*, the protein product of which, σ^D^, has been shown to affect the expression of TcdR, which, in turn, controls the expression of the large clostridial toxins, TcdA and TcdB. This demonstrates that the formation of colonies, defence from bacteriophages, cell motility and toxin production are all controlled by RecV (Anjuwon-Foster and Tamayo 2017). Two other sites likely to be inverted by RecV have also been identified but the effect of their inversion is yet to be characterised.

Five completely unrelated repeat types of approximately 80–120 residues have been identified in various ribotypes. CwpV is able to mediate aggregation and phage resistance regardless of which repeat regions it contains. Strains have been observed with between 4 and 9 repeat regions, accounting for roughly 50–75% of the residues within the protein. The five types of repeats bear no significant similarity to each other, but each show a high degree of similarity between multiple copies within a protein. The first copy of a repeat is generally afforded slightly greater sequence variability (Reynolds et al. 2011).

It has previously been observed that CwpV may undergo some form of cleavage, however it was unclear how this cleavage was mediated (de la Riva et al. 2011). Dembek et al. (2012) determined that CwpV autoproteolyses into two fragments via N-O acyl migration. The cleavage site, Gly412-Thr413, is roughly half way between the CWB2 domains and the Ser/Gly rich region. Asp411 deprotonates Thr413, which then nucleophilically attacks Gly412, forming a hydroxyoxazoladine intermediate (Dembek et al. 2012). This is reduced to an ester, and then hydrolysed to produce the cleaved products: an N-terminal product of approximately 42 kDa, and a C-terminal product of up to 120 kDa (Reynolds et al. 2011). The extreme chemical conditions normally required for N-O acyl rearrangements are believed to be made unnecessary by unusual torsion of Asp411. The mechanism was confirmed by a series of mutations (Dembek et al. 2012). The two products have been shown to co-elute, so it is likely that they form a non-covalent complex, with the highly conserved regions either side of the cleavage site potentially forming an interface between the two cleavage products (Reynolds et al. 2011). It is currently unknown if there is any similarity between this interface and the one within the H/L complex.

## Cwp19

**Fig. 7:**
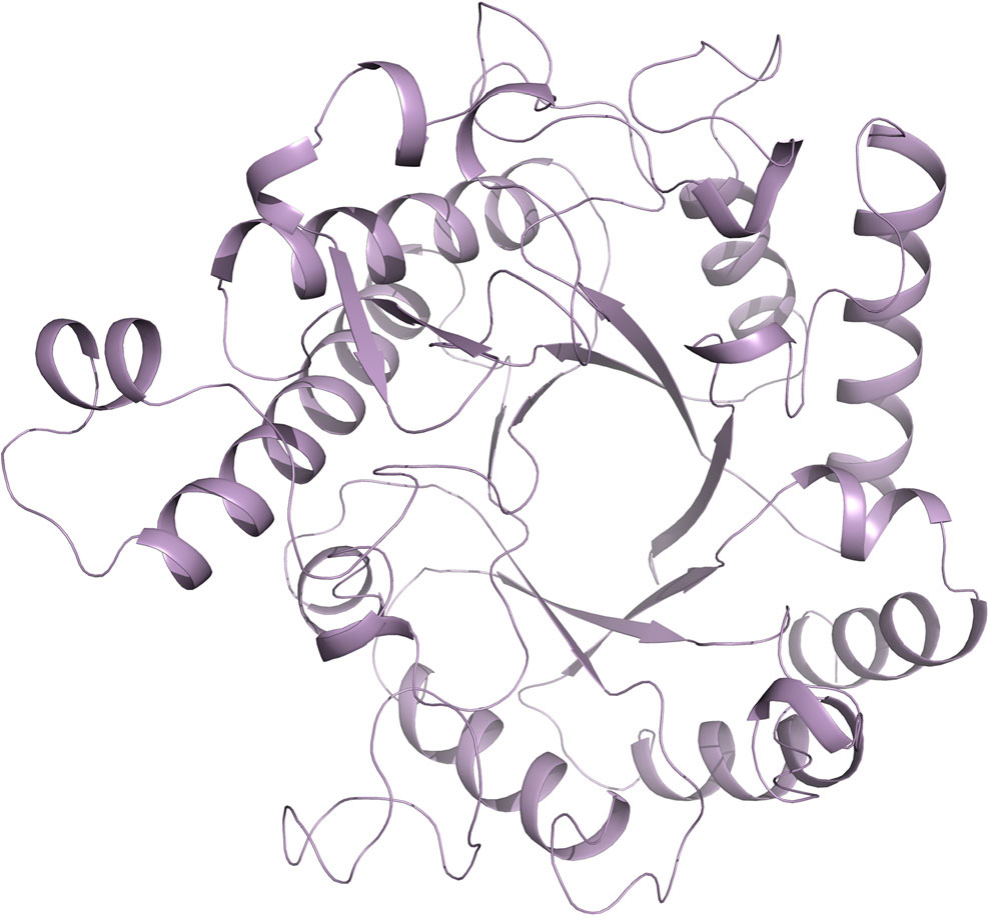
The structure of Cwp19. 5OQ2, 5OQ3 (Bradshaw et al. 2017b). The GHL10 domain of Cwp19 has a typical TIM barrel fold formed by eight β-strands surrounded by eight α-helices. The active site is located centrally over the barrel

The gene coding for Cwp19 is found within the AP locus, which has been linked to synthesis of PSII, the surface presented repeating hexasaccharide to which CWB2 domains bind (Chu et al. 2016; Ganeshapillai et al. 2008; Willing et al. 2015). Cwp19 possesses an N-terminal family 10 glycoside hydrolase-like (GHL10) domain (Naumoff 2011), so it is possible that the protein may play a role in formation of PSII. It has been shown that Cwp19 is capable of cleaving peptidoglycan (Peltier et al. unpublished results), while we have recently determined the structure of the GHL10 domain (PDB: 5OQ2, 5OQ3, Fig. 7), which assumes a TIM barrel fold, common to many glycoside hydrolases. We also showed that the peptidoglycan hydrolase activity of Cwp19 is an order of magnitude slower than that of lysozyme and that Cwp19 appears to show a high degree of substrate selectivity as it was unable to break down any other carbohydrates tested (Bradshaw et al. 2017b).

A recent study on seven *C. difficile* strains found in Brazil indicated that the amount of Cwp19 in S-layer extracts was higher than any other protein in three strains and second only to Cwp2 in two strains and SlpA in one (Ferreira et al. 2017). Such a high degree of expression would suggest an important role due to the metabolic cost of producing this amount of protein. The precise function of Cwp19 is yet to be established.

## Cwp20

Cwp20 possesses an N-terminal region of unknown structure and function of approximately 60 residues, followed by a β-lactamase domain, another region of unknown structure and function of around 320 residues and C-terminal cell wall binding domains.

β-lactamases are the most widely studied group of antibiotic resistance enzymes. They were discovered in 1940, before β-lactam antibiotics (including penicillins, cephalosporins, monobactams, carbapenems and others) entered clinical use (Abraham and Chain 1940). They now serve as the primary antibiotic resistance mechanism in Gram-negative bacteria. β-lactamases are a diverse group of antibiotic resistance enzymes; many species express several, resulting in resistance to a wide range of β-lactam antibiotics (Liakopoulos et al. 2016). There are currently 17 known β-lactamases or penicillin binding proteins coded for by the *C. difficile* genome, including Cwp20, which makes the therapeutic use of β-lactams difficult (Monot et al. 2011; Sebaihia et al. 2006).

## Cwp21 and Cwp26

Cwp21 features N-terminal cell wall binding domains followed by three PepSY domains while Cwp26 is predicted to contain one C-terminal PepSY domain separated from the CWB2 domains by an uncharacterised region of approximately 120 residues (Eddy 2008). PepSY domains, which derive their name from peptidase and *Bacillus subtilis* YpeB, are usually 60–75 residues long, are believed to act as protease inhibitors and are frequently (though not always) found in protease propeptides. Sequence conservation among PepSY domains is usually very low with only a central aromatic residue and an aspartate flanked by two hydrophobic residues with a nearby glycine residue showing a high level of conservation, although even these are not always present. It has been speculated that secreted proteins containing PepSY domains may play a role in controlling the bacterium’s environment and pathogenesis (Yeats et al. 2004).

## Cwp22

Cwp22 contains a YkuD domain followed by 8 type 1 cell wall binding (CWB1) repeats (Eddy 2008). YkuD domains, which were previously known as ErfK/YbiS/YcfS/YnhG domains, are now named after a protein from *B. subtilis*, the first in the family to have its structure determined (Bielnicki et al. 2006). YkuD domains are L,D-transpeptidases, which appear to perform roles similar to the more common D,D-transpeptidases involved in peptidoglycan crosslinking. The reversal of stereochemistry seen in L,D-transpeptidases is believed to confer resistance to β-lactam antibiotics (Biarrotte-Sorin et al. 2006). The proteins are composed of a β-sandwich and possess a conserved active site consisting of a (Y/L)XXHG(S/T) motif closely followed by SXGC(I/V)R(M/L), with the histidine, first glycine, cysteine and arginine forming a catalytic tetrad.

The 20 residue CWB1 repeats, which have been seen in a wide range of proteins from Gram-positive bacteria, assume a β-hairpin fold and contain conserved hydrophobic residues, aromatic residues and glycines (Fernandez-Tornero et al. 2001). Successive β-hairpins are orientated at approximately 120° to each other, resulting in a left-handed superhelix. CWB1 repeats are found in choline binding proteins and glucosyltransferases (Shah et al. 2004). Both the choline and the carbohydrate binding sites are formed by the interface between adjacent hairpins (Fernandez-Tornero et al. 2001). Interestingly, these repeats are also seen in the binding domain of the large clostridial toxins (Davies et al. 2011).

## Cwp24

Cwp24 has N-terminal cell wall binding domains followed by a region of unknown structure and function of approximately 60 residues and a C-terminal Glycoside hydrolase family 73 domain, specifically, an endo-β-N-acetylglucosaminidase domain. This is predicted to cleave between N-acetylglucosamine (NAG) and N-acetylmuramic acid (NAM) in peptidoglycan (Eddy 2008; Finn et al. 2016; Jones et al. 2014). This could be for remodelling of the *C. difficile* cell wall, or for attacking competing bacteria.

## Uncharacterised regions

Despite the wide range of putative domains currently identified, eight Cwps, namely, Cwp66, 5, 20, 23, 26, 27, 28, and 29 each contain regions of around 100 residues or more for which no structure or function has so far been predicted. This leaves a large number of potential functions of the S-layer still to be determined.

## SecA2

The secretory pathway is responsible for the majority of protein translocation across Gram-positive cell walls. Proteins possessing a signal peptide are passed through the SecYEG channel by the ATPase activity of SecA, frequently after recognition by the signal recognition particle (SRP), a ribonucleoprotein complex (Driessen and Nouwen 2008; du Plessis et al. 2011; Zhou et al. 2014). It was believed that bacteria possessed only one copy of each of the *sec* genes, however, in recent years, an increasing number of species have been shown to possess a second copy of *secA*, *secY*, or both. These genes are referred to as accessory *sec* genes (Feltcher and Braunstein 2012; Rigel and Braunstein 2008). They are usually not essential to the survival of the bacterium and are only responsible for a small portion of the secretosome – frequently proteins involved in pathogenicity.

A study by Fagan and Fairweather (2011) characterised *C. difficile*’s accessory *secA* gene, *secA2*, which is found in the slpA locus. It was demonstrated that neither of the SecA proteins are redundant and that SecA2 is necessary for the secretion of at least SlpA, Cwp2, Cwp66, Cwp84 and CwpV. As the S-layer is likely to be essential to viability in most strains, *secA2* knockouts, which would presumably be unable to form an S-layer, were not viable. *secA2* knockdowns, which were shown to have compromised SlpA and CwpV secretion, were viable, but severely stressed (Fagan and Fairweather 2011). This strongly indicates that the signal peptides of at least the identified Cwps, if not all, are sufficiently different to a typical signal peptide that they are unable to bind to SecA. The exact method by which SecA and the SRP recognise proteins for secretion has only recently begun to be elucidated (Grady et al. 2012; Zhou et al. 2014).

## Conclusions

S-layers are always important for the survival of the organisms that possess them (Sara and Sleytr 2000; Smarda et al. 2002). Because of this and the fact that, by their nature, they are surface exposed, their component proteins show significant potential as drug targets. If the unusually complex S-layer of *C. difficile* is to be exploited as a drug target, a comprehensive understanding of all of the proteins contained within it, and those involved in its formation, will be required. Since the discovery of the S-layer of *C. difficile* in 1984 (Kawata et al.), our understanding of it has increased, but there is still a considerable way to go before an overall model of its workings can be elucidated. Research in this expanding area of study has led to many interesting and unexpected revelations and there is no doubt that this will continue as further discoveries are made.
